# Integrating dynamic models and neural networks to discover the mechanism of meteorological factors on Aedes population

**DOI:** 10.1371/journal.pcbi.1012499

**Published:** 2024-09-27

**Authors:** Mengze Zhang, Xia Wang, Sanyi Tang

**Affiliations:** 1 School of Mathematics and Statistics, Shaanxi Normal University, Xi’an, People’s Republic of China; 2 School of Mathematics and Statistics, Shanxi University, Taiyuan, People’s Republic of China; Northeastern University, UNITED STATES OF AMERICA

## Abstract

Aedes mosquitoes, known as vectors of mosquito-borne diseases, pose significant risks to public health and safety. Modeling the population dynamics of Aedes mosquitoes requires comprehensive approaches due to the complex interplay between biological mechanisms and environmental factors. This study developed a model that couples differential equations with a neural network to simulate the dynamics of mosquito population, and explore the relationships between oviposition rate, temperature, and precipitation. Data from nine cities in Guangdong Province spanning four years were used for model training and parameter estimation, while data from the remaining three cities were reserved for model validation. The trained model successfully simulated the mosquito population dynamics across all twelve cities using the same set of parameters. Correlation coefficients between simulated results and observed data exceeded 0.7 across all cities, with some cities surpassing 0.85, demonstrating high model performance. The coupled neural network in the model effectively revealed the relationships among oviposition rate, temperature, and precipitation, aligning with biological patterns. Furthermore, symbolic regression was used to identify the optimal functional expression for these relationships. By integrating the traditional dynamic model with machine learning, our model can adhere to specific biological mechanisms while extracting patterns from data, thus enhancing its interpretability in biology. Our approach provides both accurate modeling and an avenue for uncovering potential unknown biological mechanisms. Our conclusions can provide valuable insights into designing strategies for controlling mosquito-borne diseases and developing related prediction and early warning systems.

## Author summary

When simulating population dynamics, scholars often rely on predefined functions to describe processes such as growth, development, and mortality. However, these functions may not be able to accurately capture the underlying biological mechanisms, posing challenges for related research. In this study, we adopted a data-driven approach integrated with mechanistic principles to simulate mosquito dynamics. Given the unknown functional relationships between oviposition rate, temperature, and precipitation, we employed a neural network to model these relationships, resulting in a model that combines the neural network with traditional mechanistic approaches. This method can successfully simulate the changing trends of mosquitoes, infer unknown parameters, train the neural network, and capture the relationships between oviposition rate, temperature, and precipitation. Data from nine cities in Guangdong Province were used for model training, while data from the remaining three cities were used for model validation. Our model successfully fitted data from all cities using the same set of parameters, demonstrating satisfactory performance. On this basis, we successfully identified the functional form between oviposition rate, temperature, and precipitation through the symbolic regression algorithm, which was consistent with the results obtained from the neural network. These findings show that our method can accurately simulate population dynamics while exploring unknown biological mechanisms.

## Introduction

Vector-borne diseases pose significant threats to human and animal populations, with mosquito-borne diseases being one of the primary causes of mortality in humans for centuries [1]. Aedes mosquitoes, as one of the major threats to severe outbreaks of mosquito-borne viruses, have been proven to be an effective vector of multiple arboviruses, such as dengue virus, chikungunya virus, yellow fever virus, and so on [2]. The behavior and life cycles of Aedes mosquitoes are highly sensitive to meteorological factors, such as temperature and precipitation [3–5]. Thus, investigating the impact of meteorological factors on the population dynamics of Aedes mosquitoes has always been a focus of attention for researchers. The existing research can be roughly divided into three main categories: biological experiment research, statistical model research, and mechanistic model research.

Biological experiments mainly refer to cultivating mosquitoes in a controlled laboratory environment to observe and analyze their survival and development under varying climatic conditions, such as temperature and humidity. For example, Delatte, Apperson, Brady, and other scholars have studied the effects of temperature on mosquito development, sexual nutrient cycle, the maximum number of eggs laid, and survival time of adult mosquitoes when other conditions remained unchanged [6–8]. However, such controlled conditions are difficult to reproduce in actual scenes, and the conclusions obtained from biological experiments may be challenging to validate in real environments. Statistical models are mainly based on controlled experiments or field observations to explore the correlations between mosquito abundance and meteorological factors [9–13]. Usually, the analysis of these correlations is linear, and the conclusions are obvious. However, it is difficult to precisely quantify the effects of meteorological factors like temperature and humidity on different life stages, such as development and mortality.

A common method in mechanistic models is to use differential equations to describe various stages of the mosquito life cycle, incorporating climate factors like temperature and precipitation through different forms of functions, such as oviposition rate, mortality rate, and development rate. Based on diverse research contents, scholars have divided the life cycle of Aedes mosquitoes into various stages and established differential equations of different dimensions [14–16]. For instance, Fukui et al. developed a four-stage model [17], Erickson et al. established a six-stage model [18], and the model proposed by Cailly et al. includes ten different stages [19]. However, when establishing these dynamics models, the functional forms for key parameters such as development rate, mortality rate, and oviposition rate need to be predefined, which poses a significant challenge to model establishment. Scholars have proposed some functional forms based on biological experiments, including Gaussian functions, and forms based on degree-day relationships for development rate, as well as monotonic exponential functions, and linear functions for mortality rate [9,20–26]. However, it is important to note that these functional forms are typically derived under controlled laboratory conditions and may not necessarily be applicable in natural environments. In addition, some functional forms remain unknown. For example, the oviposition behavior of mosquitoes may be influenced by both temperature and precipitation [6,27], but existing proposed oviposition rate functions often focus solely on one factor, either temperature or precipitation [9,25,28]. The precise form of oviposition rate under the combined influence of temperature and precipitation has yet to be determined.

In recent years, neural networks, distinguished by their unique network structure and information processing methods, have made brilliant achievements in many fields. They have been effectively employed in many tasks including mosquito species classification and abundance prediction. For example, Lee, Kinney, and others have successfully simulated mosquito abundance by constructing neural network models such as feedforward neural networks, long short-term memory (LSTM) networks, and gated recurrent units (GRUs), achieving ideal simulation results [1,29–32]. However, the common operation of these methods is to take factors such as time and weather as inputs, with mosquito abundance as output. This end-to-end learning method cannot reveal the biological mechanism of mosquito growth and reproduction. The black-box nature of the algorithm makes the deep learning method encounter unexplained risks [33]. In order to improve the interpretability of deep learning in physics, biology, chemistry, and other issues, more and more works pay attention to coupling or embedding differential equations and deep neural networks. It has recently been shown to be advantageous to merge differential equations with machine learning [34], such as neural differential equations and universal differential equations [35–37]. Despite the remarkable progress in coupling the differential equation and deep learning, these methods haven’t attracted enough attention and widespread application, with most existing research focusing on epidemiological dynamics problems [33,38–39]. In fact, there are many unknown mechanisms in biological growth and development processes. Introducing the idea of coupling neural networks with differential equations into population dynamics models can help us address many issues. Furthermore, despite the improved interpretability of neural networks achieved through this coupling model, it still faces criticism for its "black-box" nature, which has prompted researchers to seek exact function expressions. Some data-driven methods, such as symbolic regression and sparse recognition of nonlinear dynamical systems, provide methods to explore unknown functional forms between variables based on data [40–43]. Combining these methods with neural networks can further enhance the interpretability of the model.

In this study, we will propose a modeling framework that combines deep learning and population dynamic models, which allows the neural network to adhere to the biological mechanism of population development and reproduction throughout the training process. In the absence of explicit mathematical formulations for the relationship between oviposition behavior and temperature/precipitation, we use real data to drive the model, approximating the oviposition rate function through a neural network, and embedding it into the traditional mosquito population dynamics model. After the model training, symbolic regression is used to interpret the input and output of the neural network and to explore the explicit expression of the oviposition rate function. By combining data and mechanisms, this modeling framework can effectively simulate population dynamics while exploring some unknown biological mechanisms. This approach can offer new insights for traditional population dynamics problems.

## Method

### Data

**Fig 1:**
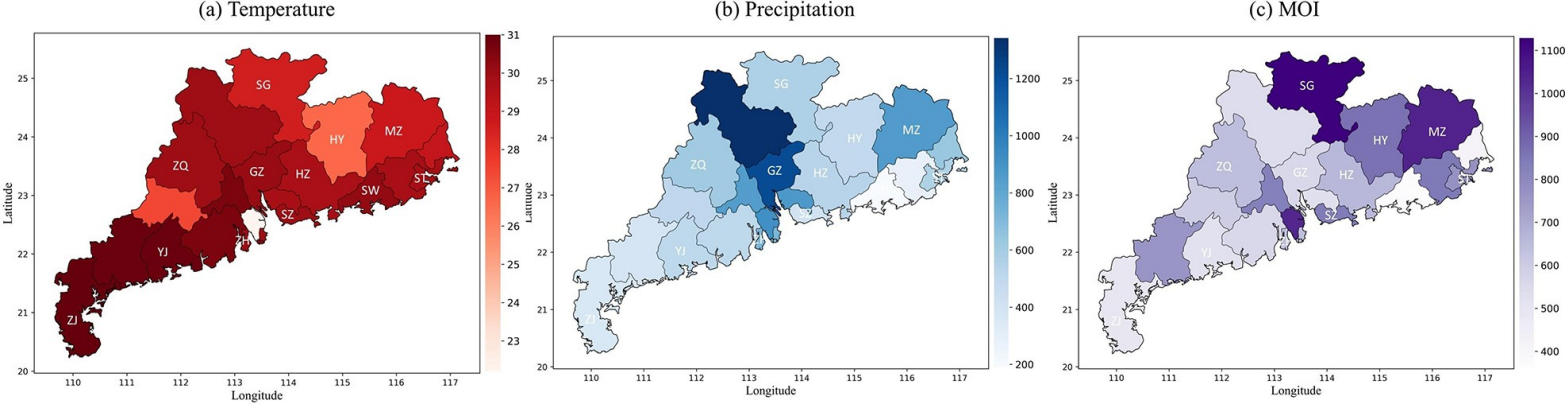
Temperature, precipitation, and Mosquito Ovitrap Index (MOI) data in Guangdong Province. (a)-(b) Average monthly temperature and cumulative monthly precipitation for all regions of Guangdong Province in June 2016. (c) MOI data for different regions of Guangdong Province during the latter half of June 2016. Darker colors indicate higher numerical values, with study areas marked by the initial letters of city names. The base layer of the map was created using the publicly available dataset ’China—Subnational Administrative Boundaries’ contributed by the Office for the Coordination of Humanitarian Affairs (OCHA) Regional Office for Asia and the Pacific. The dataset can be accessed at: https://data.humdata.org/dataset/china-administrative-boundaries. The data license can be found at: https://data.humdata.org/faqs/licenses. We affirm that no modifications have been made to the original data.

**Fig 2:**
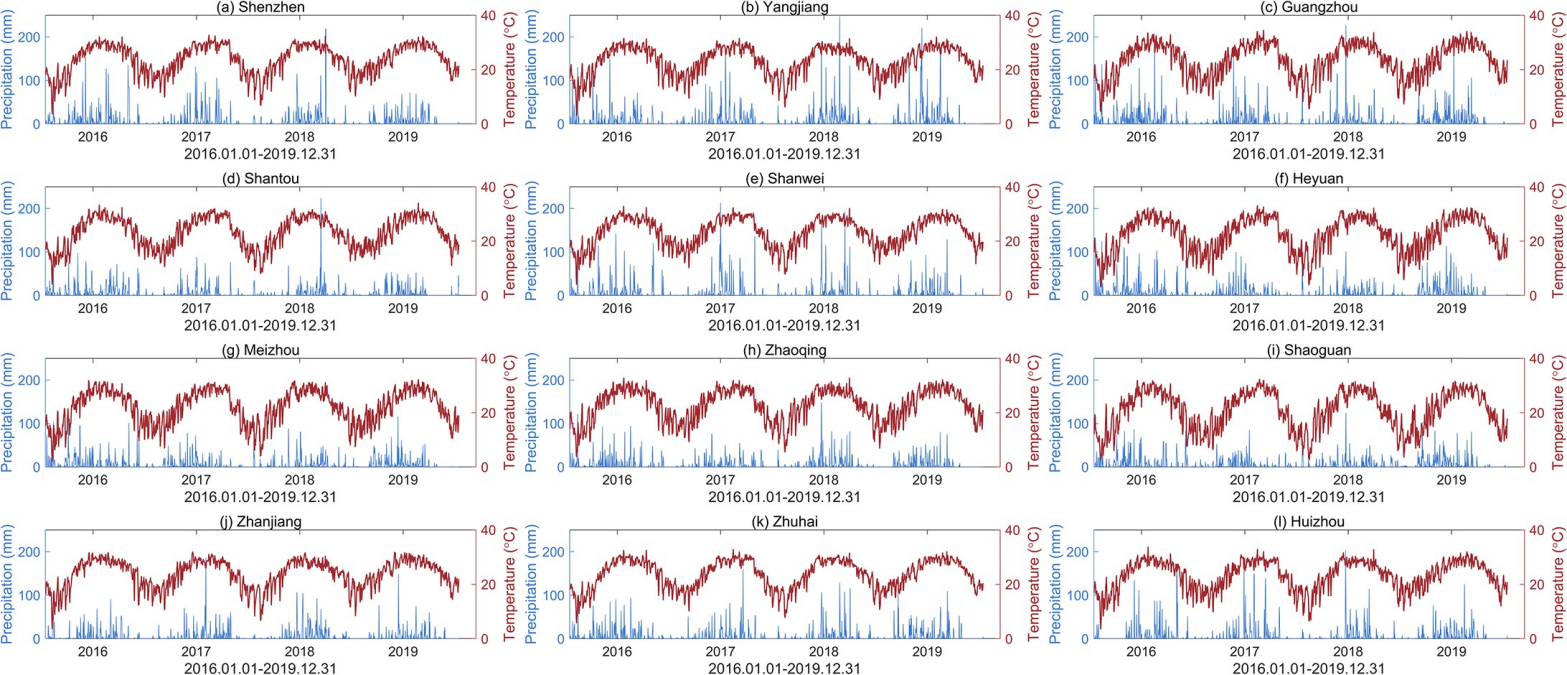
Meteorological data for twelve cities. Daily cumulative precipitation (blue) and daily average temperature (red) from January 1, 2016, to December 31, 2019, for twelve cities.

Guangdong Province is located in the southern coastal region of China and falls within the subtropical monsoon climate zone. The monthly average temperature and monthly cumulative precipitation for each city in Guangdong Province in June 2016 are shown in Fig 1A and 1B. As depicted in the figures, notable variations in temperature and rainfall among cities are observed due to geographical differences, such as disparities between the northern and southern parts of the province and the influence of mountainous and hilly terrain. These diverse climate patterns contribute to significant differences in mosquito abundance among cities (shown in Fig 1C). Based on these observations, we selected twelve representative cities—Shenzhen, Yangjiang, Guangzhou, Shantou, Shanwei, Heyuan, Meizhou, Zhaoqing, Shaoguan, Zhanjiang, Zhuhai, and Huizhou—for further analysis and research. The daily average temperature and daily cumulative precipitation of these cities can be obtained from the National Centers for Environmental Information (NCEI) website (https://www.ncei.noaa.gov), which are shown in Fig 2.

The Mosquito Ovitrap Index (MOI) of these cities, providing insights into the fluctuation of Aedes mosquito populations, was obtained from the Guangdong Provincial Health Commission (https://wsjkw.gd.gov.cn). According to the monitoring guidelines for dengue vector published by the Guangdong Provincial Health Commission, monitoring staff establish multiple monitoring points in each city to assess the populations of Aedes mosquitoes, including both Aedes albopictus and Aedes aegypti. The combined results of these two mosquito species will be used as the overall monitoring results for Aedes mosquitos. The Guangdong Provincial Health Commission reports monitoring results every half month. These reports detail the proportions of four categories of monitoring points in each city—meeting epidemic prevention and control standards, low-density, medium-density, and high-density areas—with corresponding MOI values for each category. Based on the distribution of monitoring points across different monitoring levels, we can calculate the data of 100 monitoring points for each city. In addition, considering the differences in data reporting methods before 2015 and the potential impact of the COVID-19 pandemic on the population dynamics of Aedes mosquitoes after 2020, the study period was selected from 2016 to 2019 to ensure reliability and accuracy.

### Biological mechanism model

The life cycle of Aedes mosquitoes includes three water-dependent stages (egg, larva, and pupa), and one aerial stage (adult). The development and death of each stage depend on several factors, such as temperature, or water availability. Based on the work of Gong et al. and Wang et al. [21,26], in this study, we collectively refer to the first three water-dependent stages as the immature stage, and the aerial stage as the adult stage. Combining biological mechanisms such as reproduction, development, and mortality, we establish a two-stage population dynamics model comprising immature (P) and adult stages (A):

$$\begin{array}{l} \frac{dP}{dt}=ANN(T(t),R(t))\cdot A-dp(T(t))\cdot P-mp(T(t))\cdot P\\ \frac{dA}{dt}=ef(R_{norm}(t))\cdot dp(T(t))\cdot P-ma(T(t))\cdot A \end{array}$$ (1)

Where *ANN*(*T*(*t*),*R*(*t*)) is the oviposition rate, which depends on the daily temperature and precipitation, *mp*(*T*(*t*)) is the mortality rate of immatures, *ma*(*T*(*t*)) is the mortality rate of adults, *dp*(*T*(*t*)) is the development rate from immatures to adults, and *ef*(*R*_*norm*_(*t*)) is the success rate of emergence dependent on the weekly precipitation. Parameter definitions can be found in Table 1, and these parameters are on a daily time scale.

**Table 1:** Definitions of the parameters used in the model.

Parameter	Definitions
*ANN*(*T*(*t*),*R*(*t*))	The daily oviposition rate affected by temperature and precipitation (day^-1^)
*dp*(*T*(*t*))	The daily development rate of immatures (day^-1^)
*mp*(*T*(*t*))	The daily mortality rate of immatures (day^-1^)
*ma*(*T*(*t*))	The daily mortality rate of adults (day^-1^)
*ef*(*R*_*norm*_(*t*))	The daily density-dependent success of adult emergence (day^-1^)

Many studies have revealed the significant impact of temperature on insect development, and the developmental and mortality processes of mosquitoes also exhibit temperature dependence. Therefore, the setting of mortality rates for both immatures and adults in this study is based on the temperature-dependent function proposed by Otero et al. [22], which resembles the Gaussian function. This is a common functional form for mosquitoes [21] and was used by Scholars such as Li et al. in the study of Aedes [44]. The development rate from immatures to adults follows the Sharpe & DeMichele equation proposed by Sharpe et al. [45], which was widely used for describing the development process of Aedes. [20,22–23]. The success rate for emergence is constrained by environmental carrying capacity and precipitation, with the functional form inspired by the research of Fukui et al. and Tran et al. [17,25].

Furthermore, diapause is a survival strategy adopted by organisms in adverse conditions, crucial for maintaining population size and regulating population growth [46]. There are two primary methods to integrate diapause into mosquito population models. One method is to use piecewise functions to distinguish the survival rate or development rate between normal and diapause stages, while another method treats diapause as an independent dynamic process with significantly different dynamics from the normal period. In addition, it is meaningful to further consider the incubation period before returning to the normal state after the diapause period [47]. In this study, to maintain model simplicity and avoid introducing too many parameters, we opted to follow the first method and modified the mortality and development rate functions for immature stage. Based on the work of Liu et al. [48], we set that mosquitoes enter diapause when the average temperature of half a month drops below 21 degrees and the duration of daylight falls below 13 or 14 hours. Considering the sunshine data in Guangdong Province, these sunshine conditions are usually met in winter, so we focus solely on temperature. During diapause, the mortality rate of diapausing eggs is low, enabling them to survive the long winter [49–50]. Thus, we segmented the mortality function of immature stage to maintain a low rate when the temperature is continuously below 21°C. We also adjusted the developmental rate during diapause to be *δ* times that of the normal period.

The expressions for the development rate, the mortality rate, and the emergence rate in the model are defined as follows:

$$\left\{ \begin{array}{l} mp(T(t))=\left\{ \begin{array}{l} 1-\mu_{p}\exp(-\frac{{\left(T(t)-T_{p}\right)}^{2}}{V_{p}^{2}}),T_{month}(t)\geq 21{}^{\circ}C\\ 1-\mu_{p}\exp(-\frac{{\left(21-T_{p}\right)}^{2}}{V_{p}^{2}}),T_{month}(t)<21{}^{\circ}C \end{array}\right.\\ ma(T(t))=1-\mu_{a}\exp(-\frac{{\left(T(t)-T_{a}\right)}^{2}}{V_{a}^{2}})\\ dp(T(t))=\left\{ \begin{array}{l} AA\cdot \frac{T(t)+273.15}{298.15}\cdot \frac{\exp(\frac{HA}{1.987}(\frac{1}{298.15}-\frac{1}{T(t)+273.15}))}{1+\exp(\frac{HH}{1.987}(\frac{1}{TH}-\frac{1}{T(t)+273.15}))},T_{month}(t)\geq 21{}^{\circ}C\\ \delta \cdot AA\cdot \frac{T(t)+273.15}{298.15}\cdot \frac{\exp(\frac{HA}{1.987}(\frac{1}{298.15}-\frac{1}{T(t)+273.15}))}{1+\exp(\frac{HH}{1.987}(\frac{1}{TH}-\frac{1}{T(t)+273.15}))},T_{month}(t)<21{}^{\circ}C \end{array}\right.\\ ef(R_{norm}(t))=\exp(-0.1\cdot (1+\frac{dp(T(t))\cdot P(t)}{250000\cdot (R_{norm}(t)+1)})) \end{array}\right.$$ (2)

Definitions of the parameters are shown in Table 2.

**Table 2:** Definitions of the parameters.

Parameter	Definition	Value	Source
*μ*_*p*_	The survival rate at *T*_*p*_	1.0000	Estimated
*T*_*p*_	The optimum survival temperature of immatures	20.7896	Estimated
*V*_*p*_	The variance for immatures	17.4774	Estimated
*μ*_*a*_	The survival rate at *T*_*a*_	0.6000	Estimated
*T*_*a*_	The optimum survival temperature of adults	23.3492	Estimated
*V*_*a*_	The variance for adults	19.3648	Estimated
*AA*	The development rate in the absence of temperature inactivation of key developmental enzymes	0.0150	Estimated
*HA*	The enthalpy of activation of enzyme-catalyzed reactions	28116.4131	Estimated
*HH*	The change in enthalpy associated with high temperature inactivation of enzymes	35378.2344	Estimated
*TH*	The temperature at which 50% of the enzyme is inactivated by high temperature	301.6750	Estimated
*δ*	The ratio of the developmental rate between diapause and non-diapause stages.	0.0023	Estimated
*T*_*month*_(*t*)	The average temperature over half a month		Data
*R*_*norm*_(*t*)	Standardized weekly cumulative precipitation		Data

### Neural network component

In model (1), *ANN*(*T*(*t*),*R*(*t*)) is the oviposition rate, which represents the average number of eggs laid by each mosquito after finding a breeding site per unit time. Mosquito oviposition behavior is influenced by both temperature and precipitation. Precipitation increases surface humidity, providing breeding habitats and stimulating mosquito oviposition [27], while temperature affects the mosquito’s gonotrophic cycle, resulting in significant differences in maximum egg production at different temperatures [6]. Most previous studies have proposed oviposition rate functions that only consider temperature or precipitation individually [9,12,18]. Furthermore, when considering precipitation factor, more scholars choose to integrate it with the processes of egg hatching and development [25,51]. To explore the combined effects of temperature and precipitation on oviposition rates and make the oviposition rate function more realistic, we utilize a neural network to replace traditional oviposition rate and establish a model that couples neural network with differential equations.

We define an L-layer deep feedforward network, $L^{L}(U):R^{d_{input}}\to R^{d_{output}}$, where *U* represents the inputs. Let *N*^*k*^ denotes the number of neurons of the *k* th layer, then the number of neurons of the input and output layers are *N*_0_ = *d*_*input*_,*N*_*L*_ = *d*_*output*_. As we aim to explore the combined effects of temperature and precipitation on oviposition rate variation, the inputs to the neural network are temperature and precipitation: *U* = (*T*, *R*), the output is oviposition rate: *ANN*(*T*, *R*), then *d*_*input*_ = 2,*d*_*output*_ = 1. Define the weight matrix and bias vector corresponding to each layer as *W*^*k*^ and *b*^*k*^, and the activation function as *σ*, then the architecture of the neural network can be recursively represented as follows:

$$\begin{array}{l} -Input:U(T,R)\in R^{2},\\ -Input\;layer:L^{0}(U)=U,\\ -Hidden\;layers:L^{k}(U)=\sigma (W^{k}L^{k-1}(U)+b^{k})\in R^{N_{k}},for\;k=1:L-1,\\ -Output\;layer:L^{L}(U)=\sigma (W^{L}L^{L-1}(U)+b^{L})\in R,\\ -Output:ANN(T,R)=L^{L}(U) \end{array}$$

Theoretically, a two-layer neural network is sufficient to fit any bounded continuous function. In practice, after multiple iterations and adjustments, we finally constructed a three-layer feedforward network, that is *L* = 3, and the number of neurons in the hidden layer are *N*_1_ = 10, *N*_2_ = 10. According to the universal approximation theorem [52–53], this is enough to approach the oviposition rate function. We choose the Sigmoid function as the activation function and map the output of the network to lie between the maximum oviposition rate and the baseline rate, which is derived from the conclusion of Liu et al., Tran et al., and Metelmann et al. [25,48,51].

**Fig 3:**
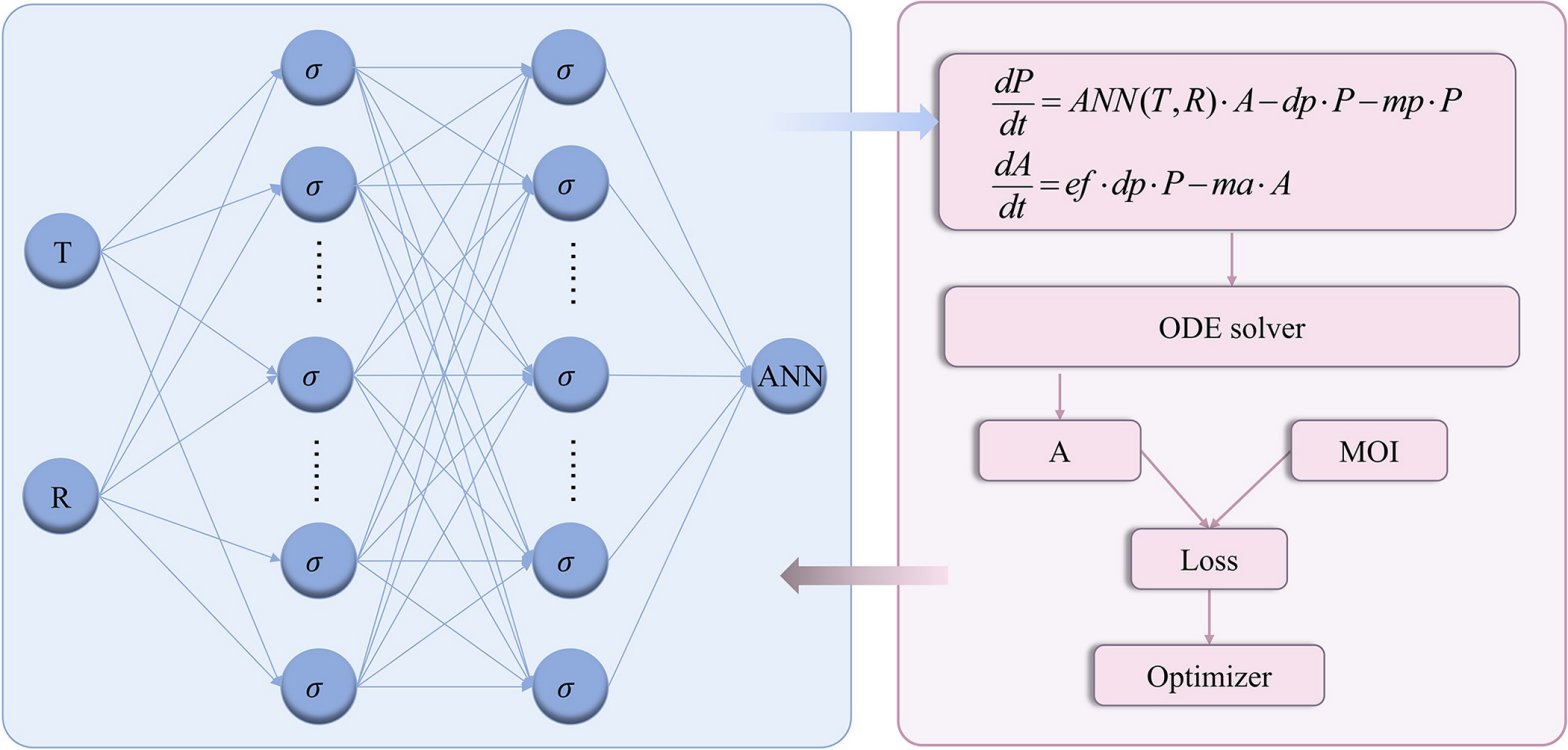
Schematic diagram of the coupling model of neural network and differential equations. The blue shading represents the neural network, and the red shading represents the mechanistic model. The output of the neural network serves as an internal dependency of the differential equation, and the neural network is indirectly trained through the differential equation.

After constructing and initializing the network, the next key step is to embed the network into the mechanistic model. The essence of this coupled model is described in Fig 3. The equation comprises two parts: one part describes the mechanisms of the growth, development, and reproduction of Aedes mosquitoes based on traditional single-population dynamical system theory, while the other part depicts the unknown mechanism, specifically the relationship between oviposition rate, temperature, and precipitation. The neural network’s output serves as an internal dependency for the numerical solution of the differential equation. By employing suitable optimization methods to minimize the error between the numerical solution of the equation and the observed values, we can indirectly constrain the output of the neural network and realize the training of the network. This method emphasizes the connection between biological mechanisms and neural networks, integrates data with mechanisms, and plays a crucial role in exploring unknown mechanisms.

### Symbolic regression for interpreting the coupling term

In Model (1), we employ a neural network to explore the unknown mechanisms between oviposition rate, temperature, and precipitation. However, due to the complex internal structure and numerous parameters of neural networks, the coupling term remains as a black-box, lacking specific mathematical expressions. In recent years, some data-driven methods such as symbolic regression have been proposed to address this interpretability issue. Symbolic regression is a type of regression analysis that searches the space of mathematical expressions to find the model that best fits a given dataset, both in terms of accuracy and simplicity, without requiring any prior domain expertise or predefined underlying regression structures [54].

We traverse the temperatures from 10°C to 35°C and precipitation from 0 mm to 200 mm, generating a set of two-dimensional arrays. These arrays serve as inputs to the neural network ℒ^L^(*U*) to compute the corresponding oviposition rates. Subsequently, this dataset will be utilized as training data for symbolic regression.

The purpose of symbolic regression is to find the best function expression that can approximate the results of the neural network. Note that the result of symbolic regression is the function *SR*(*T*,*R*):*R*^2^→*R*, then the fitness function can be defined as:

$$MSE=\sqrt{\frac{1}{m\cdot n}\sum_{i=1}^{m}\sum_{j=1}^{n}{\left(SR(T_{i},R_{j})-ANN(T_{i},R_{j})\right)}^{2}}$$ (3)

Where *m* and *n* represent the sample size.

**Fig 4:**
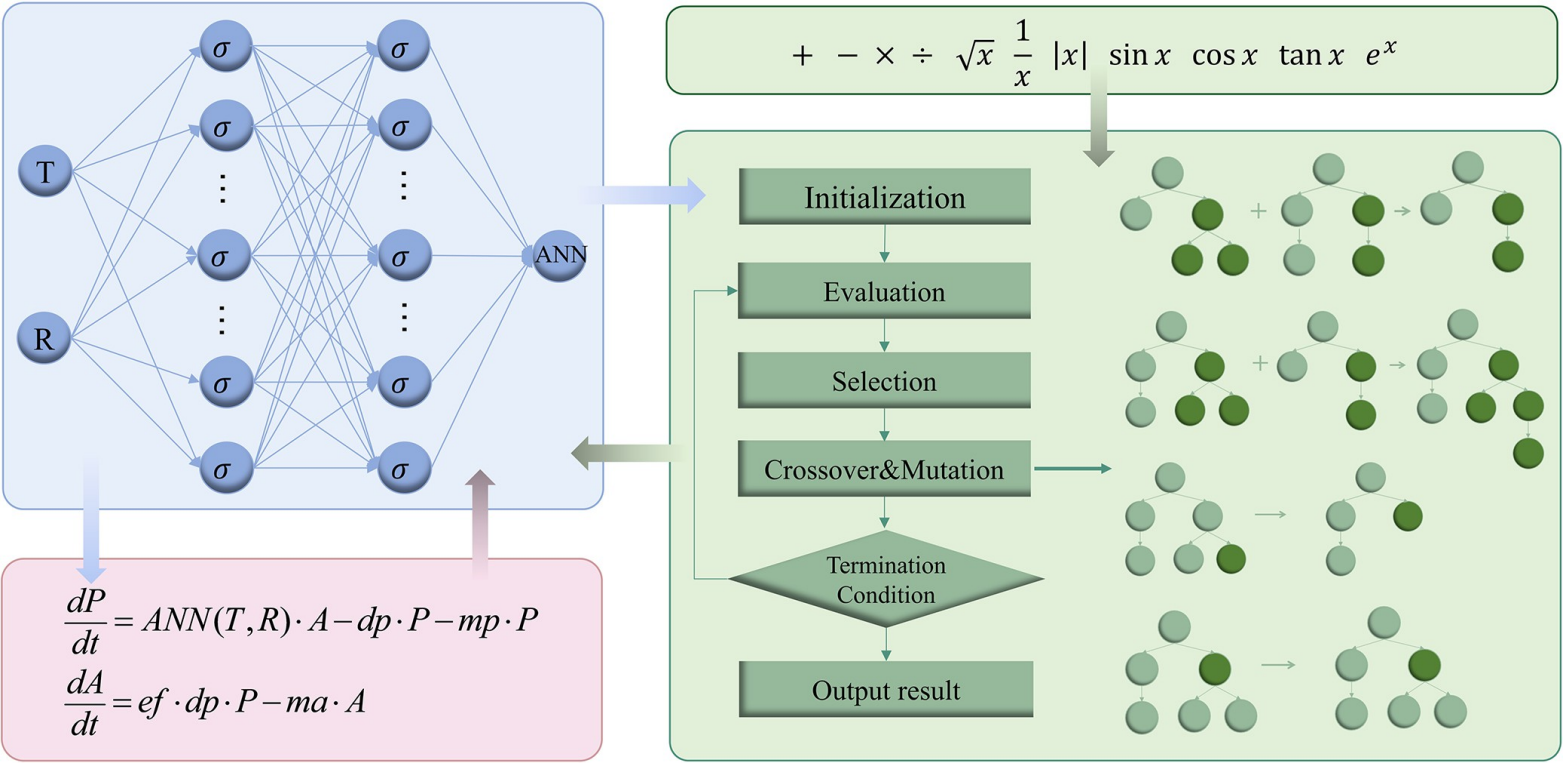
Symbolic regression flow diagram. The blue shading represents the neural network, the red shading represents the mechanistic model, and the green shading represents the process of symbolic regression using the idea of the genetic algorithm. The training data of symbolic regression is obtained according to the input and output of the neural network, and the optimal expression is found through a series of genetic, variation, and evolutionary operations.

Using the training data obtained from the above operations, with the goal of minimizing this fitness function, we employ symbolic regression to seek explicit expressions among variables, as shown in Fig 4. Genetic programming is a specific implementation of this procedure. Its core idea is to simulate natural selection, genetic variation, and other phenomena in biological systems following Darwin’s theory of evolution, evolve the solution to a given problem, and find the most suitable solution after several generations. This approach utilizes a binary tree structure to describe function expression and applies string-based genetic operations to manipulate the binary tree [55–57]. The process begins with the initial binary trees generated randomly. After generations of reproduction and evolution, we can obtain the optimal expression.

### Parameter estimation

Based on the study of Tran et al. [25], we assumed that there are 1000000 diapause eggs at the initial moment to simulate the dynamics of Aedes mosquito population. To mitigate the impact of initial conditions on simulation results, simulations commenced on January 1, 2015, and spanned five years in daily units until 2019. Data from 2016 to 2019 were utilized to constrain the parameters. The dataset was split into two segments: data from nine cities—Shenzhen, Yangjiang, Guangzhou, Shantou, Shanwei, Heyuan, Meizhou, Zhaoqing, and Shaoguan—served as training set for model development and parameter estimation, while data from three cities—Zhanjiang, Zhuhai, and Huizhou—were used as testing set to evaluate model generalization.

During the model training process, we simultaneously calculated the coupling models of the nine cities in the training set using the same set of parameters, and evaluated the overall loss function. It should be noted that the MOI doesn’t directly reflect the exact count of mosquitoes, rather, it is a commonly used index to represent the abundance of adults, which is proportional to the actual number. In fact, directly measuring the real number of mosquitoes is challenging, so we pay more attention to the changing trend in mosquito population dynamics. Our goal of parameter estimation is to align the fitted trends of adult mosquitoes with the changes in MOI. Since the MOI is published half-monthly, we calculated the average simulation results every half-month and adjust them using rotation factors to align their scales with MOI. Our definition of the loss function is

$$Loss(\theta_{1},\theta_{2})=\frac{1}{m\cdot n}\sum_{j=1}^{m}\sum_{t=1}^{n}\left|MOI_{j,t}-\lambda_{j}A_{j,t}(\theta_{1},ANN_{\theta_{2}}(T_{j,t},R_{j,t}))\right|$$ (4)

Where *θ*_1_ represents the biological parameters and *θ*_2_ represents the network parameters, *m*, *n* represent the number of cities and months, *λ*_*j*_ presents the rotation factor of city *j*.

We employed local adjoint sensitivity analysis [36] to minimize the loss function and utilized the adaptive moment estimation algorithm (ADAM) to update the parameters. The parameters to be estimated can be mainly divided into two categories. The first category comprises parameters related to the established known mechanisms (*θ*_1_), including these unknown parameters in the development rate and mortality function, as shown in Table 2. The second category is the parameters involved in the neural network (*θ*_2_), which are used to adjust the connection strength and activation rules between neurons. Notably, the same parameter set is shared across the nine cities in the training set, indicating a level of parameter generalization achieved in our study. Considering the numerous internal parameters in the neural network and the associated training costs, we implemented a three-stage optimization approach to search for the best parameters:

- Step 1: Set the parameters in sets *θ*_1_ and *θ*_2_ as trainable and adjustable parameters. Utilize the ADAM optimizer with an initial learning rate of 0.01 and employ the TRBDF2 method to solve the differential equation. Iterate the optimization process 500 times to estimate all parameters;
- Step 2: Fix the parameters of the mechanistic model (*θ*_1_) with the optimal parameters obtained in step 1. Set the parameters in set *θ*_2_ as trainable and adjustable parameters. Utilize the ADAM optimizer with an initial learning rate of 0.001, and iterate the optimization process 2000 times to estimate the network parameters *θ*_2_.
- Step 3: Fix the parameters in the neural network (*θ*_2_) with the optimal parameters obtained in step 2. Set the parameters in the set *θ*_1_ as trainable and adjustable parameters. Set the initial learning rate of the ADAM optimizer to 0.01, and perform iterative optimization 1000 times.

After the above training, a well-trained neural network can be constructed. Next, we employed symbolic regression to explore explicit expressions between the network inputs (temperature and precipitation) and outputs (oviposition rate). The training dataset for symbolic regression was constructed as follows: temperature values were sampled at 1°C intervals from 15°C to 35°C, while precipitation values were selected based on different ranges: from 0mm to 30mm at 1 mm intervals, from 31mm to 150mm at 10mm intervals, and from 150mm to 200mm at intervals of 5mm. This selection method was determined based on the observed curve shapes of the oviposition rates generated by the neural network. Details on the hyperparameters and their respective values used during the symbolic regression are listed in Table 3. The termination condition was set such that the error of the algorithm running to the optimal model is less than 0.01% or it runs to the 40th generation. The population size was set to be 6000, and the depth of individuals in the initial population varied randomly between 2 and 6. During the evolutionary process, we controlled the occurrence of point mutations in winning individuals with a probability of 0.1, hoist mutations with a probability of 0.07, and subtree mutations with a probability of 0.1. The probability of crossover among winners was set to 0.1. To control the complexity of the formula, several attempts were made to set the parsimony coefficient from 0.001 to 0.1, and it was finally determined to be 0.02.

**Table 3:** Hyperparameter in symbolic regression process.

Hyperparameter	Definition	Value
population_size	The number of individuals retained in each generation	6000
generations	The number of iterations for the algorithm	40
stopping_criteria	The stopping criteria for the algorithm	0.01
p_crossover	The probability of crossover operation occurrence	0.1
p_subtree_mutation	The probability of subtree mutation operation occurrence	0.1
p_hoist_mutation	The probability of hoist mutation operation occurrence	0.07
p_point_mutation	The probability of point mutation operation occurrence	0.1
parsimony_coefficient	Controls the complexity of the generated expressions, balancing model fit and simplicity	0.02

## Result

### Fitting results

**Fig 5:**
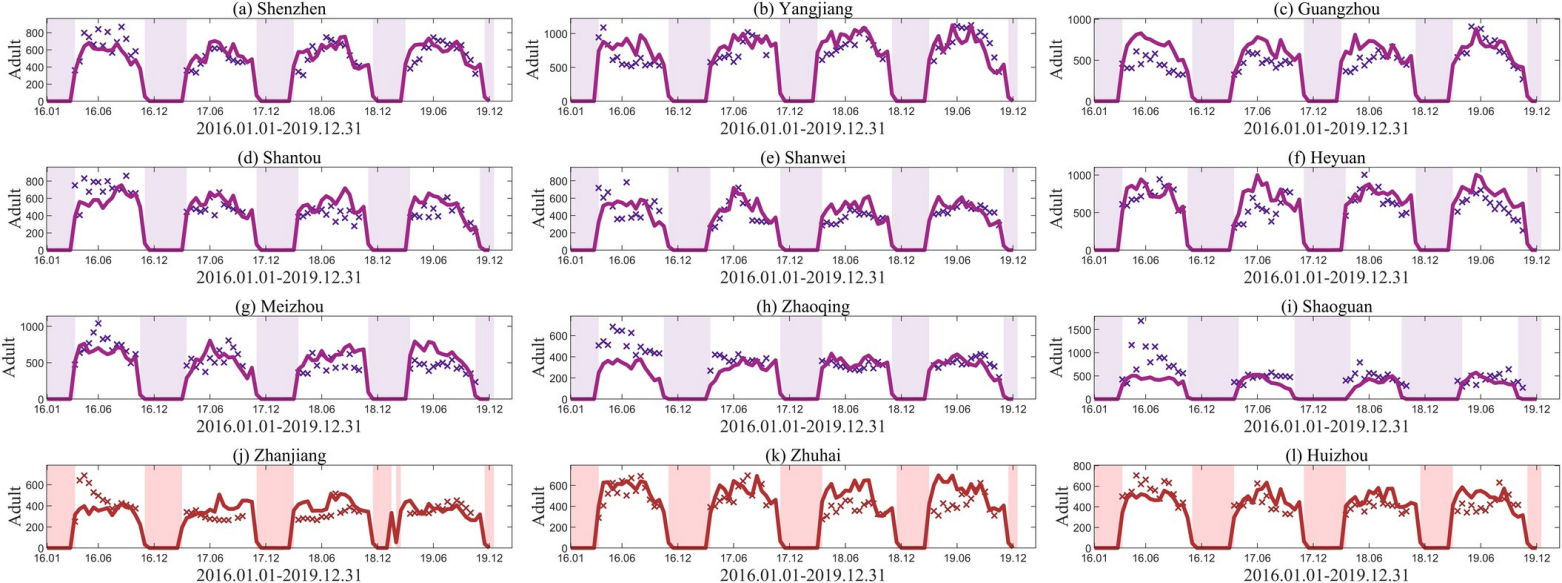
Data fitting and verification results. (a)-(i) Fitting results of adults from January 2016 to December 2019 in the training set. Purple solid lines represent the fitting results, dots indicate the training set data, and the shaded areas indicate the diapause periods. (j)-(l) Prediction results of adults from January 2016 to December 2019 for three cities in the testing set. Red solid lines represent the predicted results, dots denote test set data, and shaded areas indicate periods of diapause.

We utilized the established model to simulate mosquito populations in nine cities from the training set. To facilitate comparison and presentation, we calculated the mean of the simulated results for each half-month and standardized them to the same scale as the MOI. The optimal fitting results for adults across these nine cities were presented in Fig 5A–5I, where purple solid lines represent the fitting results, purple dots denote the training data, and shaded areas indicate periods of diapause. To evaluate the model’s generalization ability and prevent overfitting, we further extended the trained model to predict mosquito populations in the testing set cities—Zhanjiang, Zhuhai, and Huizhou—as depicted in Fig 5J–5L, where red solid lines represent the predictions and red dots denote the testing data.

**Fig 6:**
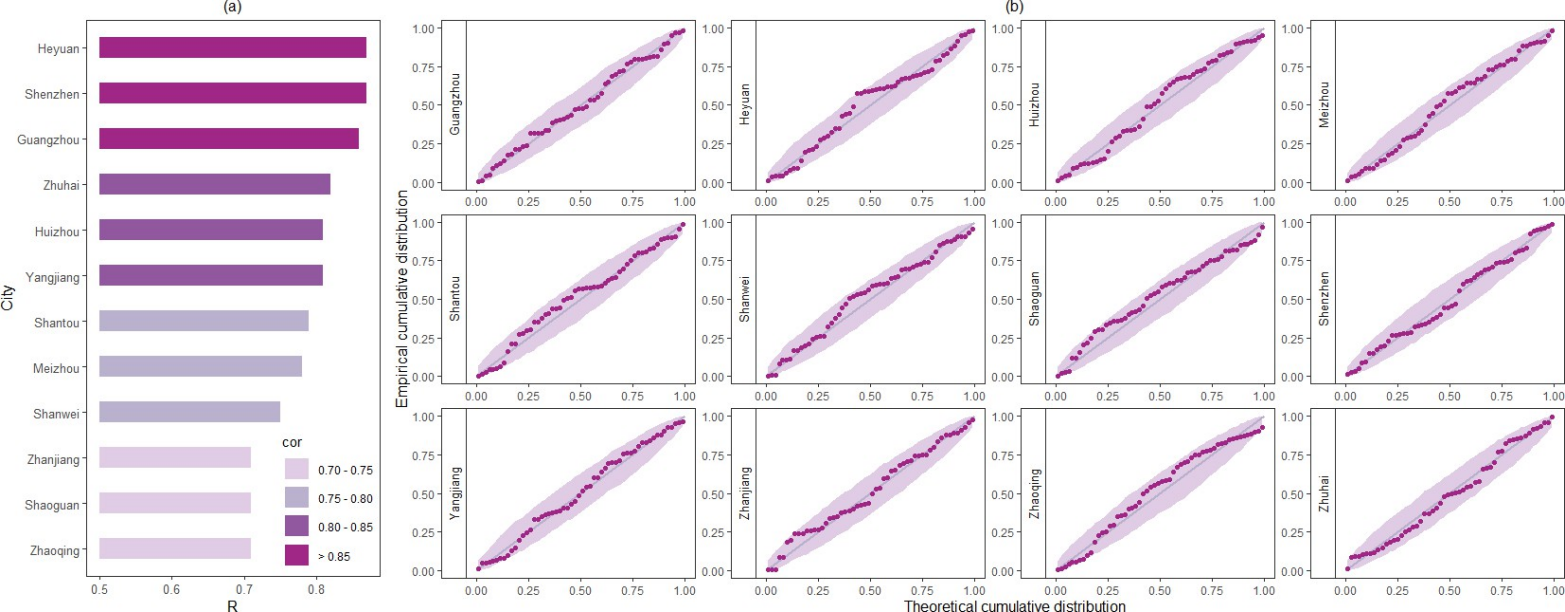
Correlation analysis and residual analysis. (a) The correlation coefficient between the simulation results and the actual values in twelve areas. The correlation coefficient is distinguished by the bar length and color depth. (b) The Probability-Probability (P-P) plots across twelve cities to assess the goodness-of-fit of residual distributions to the theoretical normal distribution. The x-axis in each plot denotes the theoretical cumulative probabilities under the normal distribution, while the y-axis represents the empirical cumulative probabilities derived from the residuals of each city. The shaded area around the diagonal dashed line represents the 95% confidence band.

It can be seen that our simulated results are closely consistent with the actual MOI in both the training and testing sets, indicating that our model effectively captures the trend of Aedes mosquito population dynamics. To further verify the fitting accuracy, the correlation coefficient (R) was used to compare the fitting values with the MOI (shown in Fig 6A). The analysis revealed that the correlation coefficients for all regions were above 0.7. Specifically, in the training set, the correlation coefficient surpassed 0.85 in Shenzhen, Guangzhou, and Heyuan, exceeded 0.8 in Yangjiang, and was above 0.75 in Shantou, Meizhou, and Shanwei City. In the testing set, the correlation coefficients of Zhuhai and Huizhou exceeded 0.8. The strong correlations observed between the simulation results and the actual values for these cities, along with their high statistical significance (P<0.01), provide robust evidence for the reliability of the simulation outcomes.

It is noteworthy that using the same set of parameters to fit data across all regions inevitably leads to a loss of local fitting accuracy. Additionally, variations exist in the standardization and accuracy of data monitoring procedures among different cities, compounded by the influence of mosquito control measures, collectively contributing to heightened data uncertainty. Considering the inherent uncertainty and randomness of the MOI, we also conducted a normality test on the residuals of the simulated results to account for them. Probability-Probability (P-P) plots were constructed to assess the goodness-of-fit of residual distributions across twelve cities to the theoretical normal distribution (shown in Fig 6B). Each panel in the figure represents a P-P plot for a specific city, comparing the observed cumulative probabilities of residuals with those expected under the normal distribution. The alignment of points along the diagonal dashed line in each panel indicates good agreement between observed and expected cumulative probabilities, suggesting that the normal distribution assumption is reasonable for modeling the residuals in these cities, thus providing additional evidence to support the validity of our model.

### Analysis of oviposition rate via the neural network

**Fig 7:**
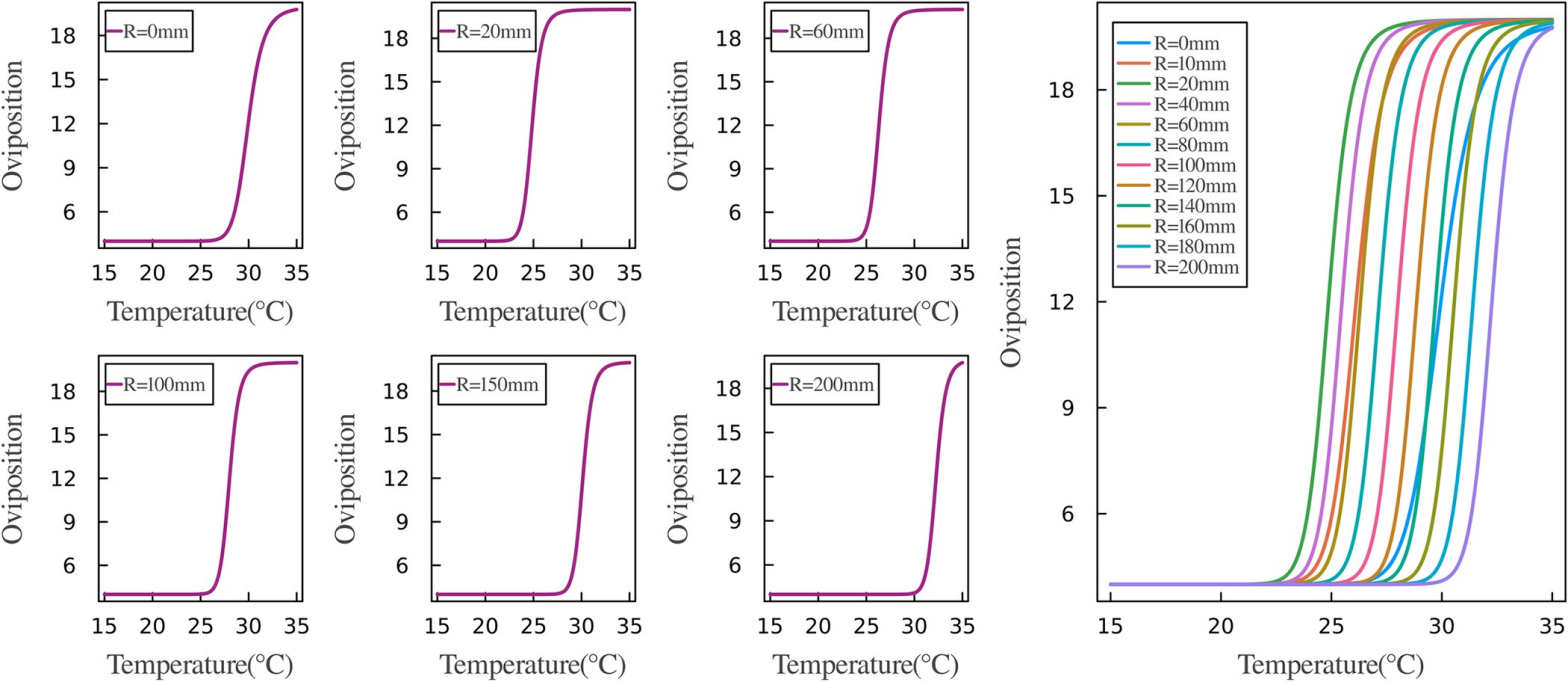
Oviposition rates fluctuating with temperatures variations. To enhance observation and analysis, the left plot illustrates the variation in oviposition rate with temperature across different precipitation levels: 0, 20, 60, 100, 150, and 200 mm. Conversely, the right plot consolidates all findings for comparative analysis, employing distinct colors to differentiate between oviposition rates at varying precipitation levels.

**Fig 8:**
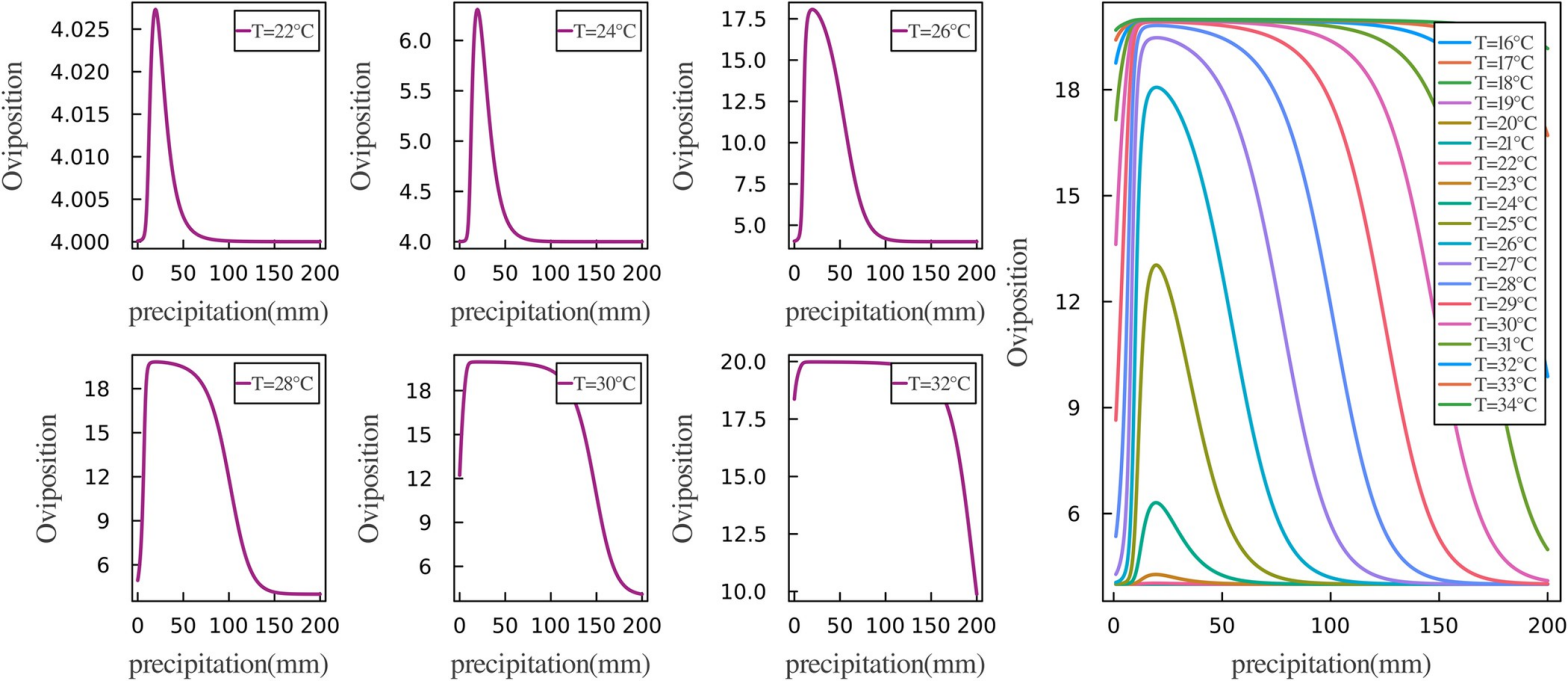
Oviposition rates fluctuating with precipitation variations. The left plot depicts the fluctuation of oviposition rates in response to precipitation across temperatures ranging from 22°C to 32°C. Conversely, the right plot consolidates all data points, with distinct colors to denote varying temperatures for ease of comparison.

Through the optimization method mentioned earlier, we obtained a set of optimal parameters. Notably, we did not provide any prior information regarding the relationships between oviposition rates, temperature, and precipitation in the model, meaning we did not assume a specific functional form for these interactions in advance. However, the trained network provided valuable insights into how oviposition rates fluctuate in response to changes in temperature and precipitation. Figs 7 and 8 show the results derived from our neural network.

The pattern of oviposition rates at various temperatures is shown in Fig 7. The figure illustrates that under constant precipitation levels, the oviposition rate follows an S-shaped curve in response to temperature changes. At lower temperatures, the oviposition rate remains low. As the temperature gradually rises beyond a certain threshold, the oviposition rate increases rapidly. However, when the temperature becomes too high, the rate of increase slows down and eventually levels off. Comparing the curves under different levels of precipitation, we observed distinct temperature thresholds for rapid increases in oviposition rate. When precipitation is either too low or too high, a temperature exceeding 25°C is required for a significant increase in oviposition rate. Under excessive precipitation, even a temperature of 30°C results in a low oviposition rate. However, at optimal precipitation levels, the oviposition rate begins to rise quickly at temperatures as low as 23°C. This curve pattern leads to fewer mosquitoes in winter and higher populations in summer and fall, which aligns with mosquitoes’ natural behavior [58]. The results demonstrate that the neural network can effectively model the relationship between oviposition rate and temperature.

The relationship between oviposition rate and precipitation variation is depicted in Fig 8. The curves representing oviposition rates at various temperatures exhibit a characteristic parabolic shape, rising initially and then falling. This pattern indicates that as precipitation levels increase, the oviposition rate also rises and maintains high values at optimal precipitation levels. However, once precipitation surpasses a certain threshold, the oviposition rate will begin to decline. Notably, the parabolic shapes vary significantly at different temperatures. The initial values of the curves differ across different temperatures, with lower temperatures associated with lower initial oviposition rates. For example, at 22°C and 24°C with low precipitation, the oviposition rate is approximately 4, but when the temperature rises to 30°C, the oviposition rate can be as high as 12. Additionally, the peak values and range of variation in oviposition rates also differ with temperature. Specifically, at lower temperatures, changes in oviposition rate in response to varying precipitation are minimal. For instance, at 22°C, the oviposition rate increases by only around 0.025 at its peak, remaining close to 4. However, at more favorable temperatures like 28°C, the range of variation in oviposition rate can exceed 12, with peak rates reaching up to 19. Furthermore, when analyzing the precipitation threshold at which the oviposition rate begins to decline significantly after reaching its peak, we found that higher temperatures extend the range of precipitation that kept the oviposition rate near its peak. For instance, at 26°C, the oviposition rate peaks at around 15mm of precipitation and subsequently decreases as precipitation continues to increase. However, at 30°C, the oviposition rate peaks when precipitation is around 10mm and remains stable until precipitation exceeds 100mm before a noticeable decrease occurs. At 32°C, oviposition rate remains high throughout a precipitation range of 10-150mm.

While previous studies generally suggested that precipitation has a positive impact on mosquito populations [59–61], some researchers argue that excessive rainfall may have negative impacts, as it may wash away mosquito habitats, affecting oviposition, egg hatching, and other factors, which in turn affect the adult mosquito populations [12,62–63]. Our study, combining biological mechanisms and neural networks, concluded that moderate precipitation positively influences mosquito populations, while excessive precipitation can have negative effects.

In summary, our results elucidate the fluctuations in oviposition rates resulting from the combined effects of temperature and precipitation. These findings are consistent with observed biological phenomena, emphasizing the efficacy of our integrated model comprising neural networks and mechanistic principles. Our coupled model not only achieves a robust fit to observed data but also enhances our understanding of the underlying biological processes.

### Oviposition rate function from symbolic regression

Through the trained neural network, we plotted the curve of oviposition rates varying with temperature and precipitation. However, the network does not provide explicit mathematical expressions. To further investigate the relationship between oviposition rate, temperature, and precipitation, and to enhance the interpretability of deep learning results, we conducted symbolic regression on the results of the neural network. The methods and parameter settings for symbolic regression are thoroughly detailed in the Methods section.

**Fig 9:**
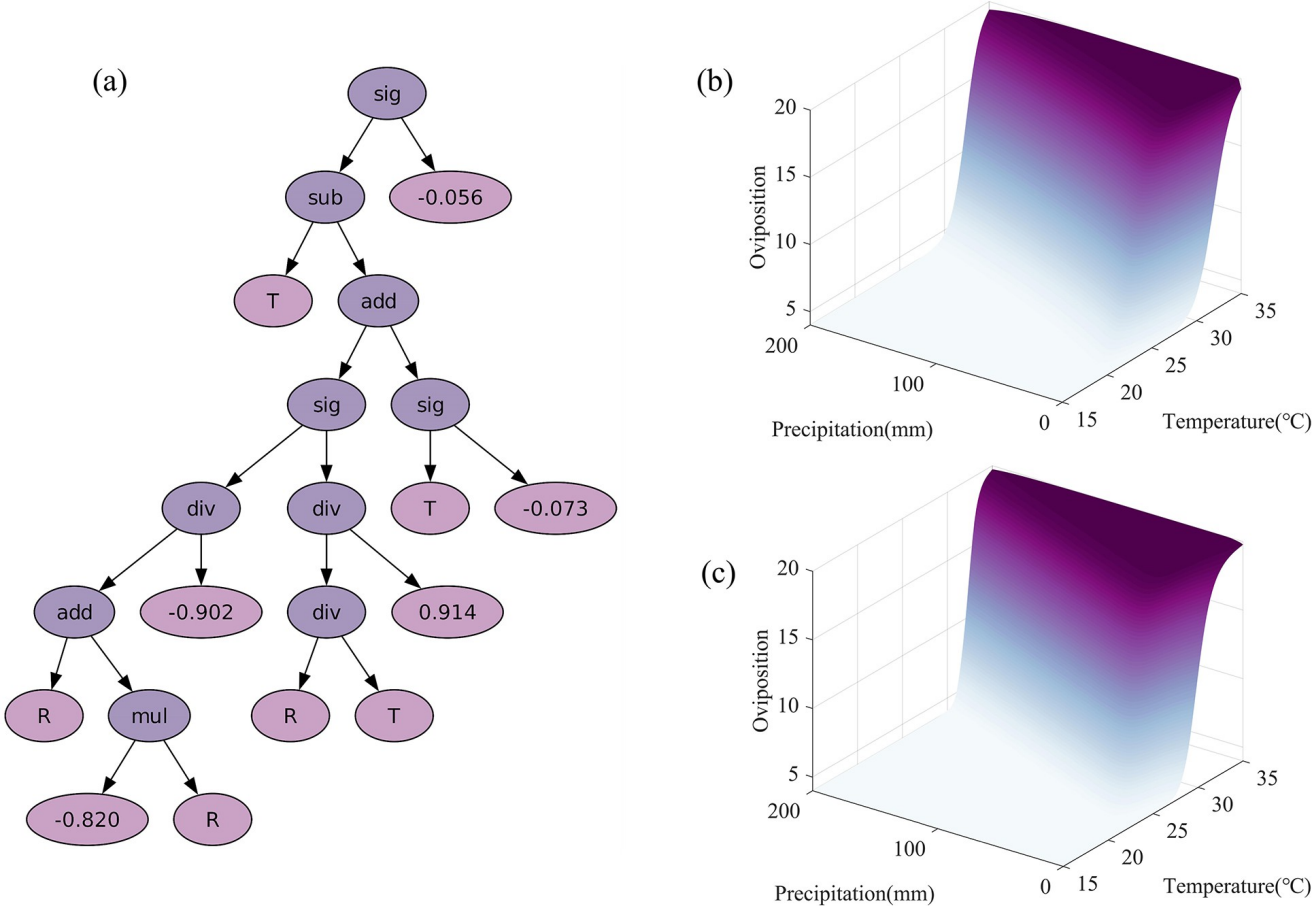
Results from symbolic regression. (a) The optimal binary tree obtained from symbolic regression using genetic programming, "sig" denotes a sigmoid function, "sub" denotes subtraction, "add" denotes addition, "mul" denotes multiplication, and "div" denotes division. (b)-(c) represent oviposition rates calculated using the neural network and symbolic regression under different temperatures and precipitation, respectively.

The binary tree obtained through symbolic regression is shown in Fig 9A. Traversing the binary tree using a depth-first algorithm and combining the mathematical operators at each node with the values of the leaf nodes, the optimal symbolic expression can be constructed. After further simplifying and optimizing, the final expression for oviposition rate with respect to temperature and precipitation is derived as follows:

$$\begin{array}{l} ANN(T,R)\approx \frac{16}{1+e^{-\phi }}+4,\\ \phi =T-(\frac{16-1.3593\frac{R}{T}}{1+e^{0.3427R}}+\frac{16.0729}{1+e^{-1.0742T}}+1.3593\frac{R}{T}+7.9271) \end{array}$$ (5)

To validate the accuracy and assess the generalization capability of this expression, we compared the oviposition rates calculated by the neural network and Eq (5) across a broader input space (temperature ranging from 15°C to 35°C at 0.5°C intervals and precipitation ranging from 0mm to 200mm at 1mm intervals). The results shown in Fig 9B and 9C exhibit a high degree of similarity between the two surfaces in patterns and fluctuations. Furthermore, we calculated the fitness function (3) for the whole spatial domain to be 1.51, providing further evidence that the derived expression effectively captures the trends in oviposition rate variations.

**Fig 10:**
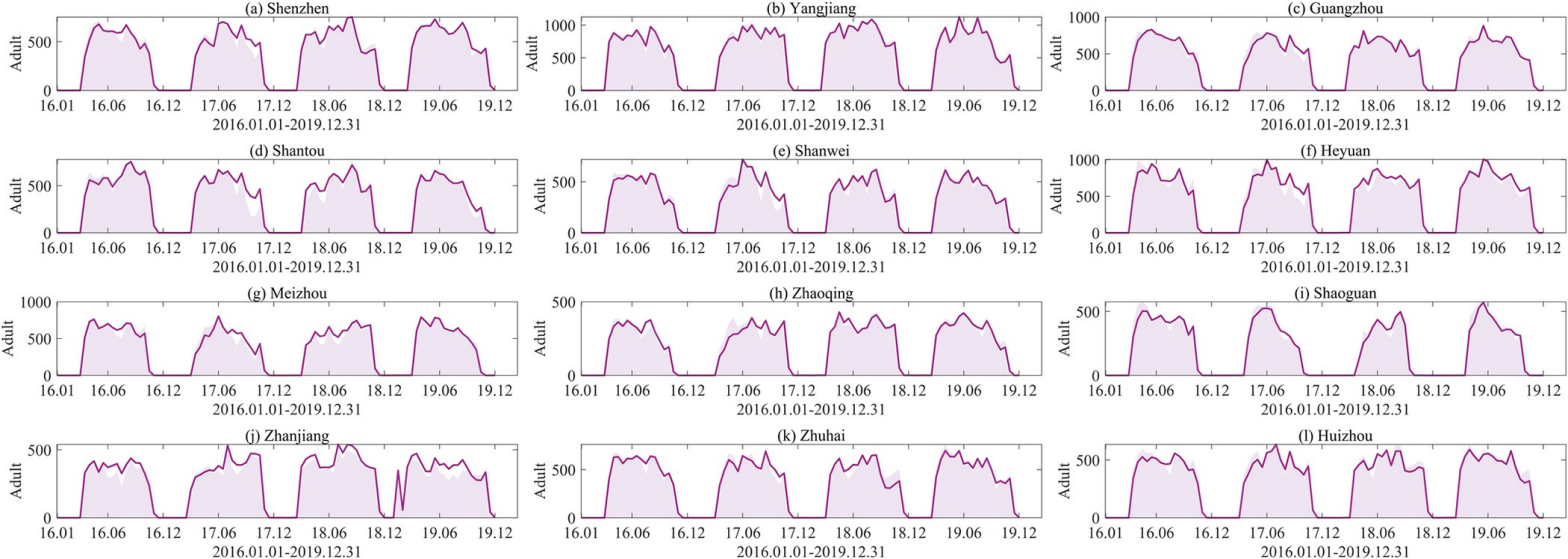
Comparison of simulation results. The shaded part represents the simulation result of using a neural network to approximate the oviposition rate function, and the solid line represents the simulation result of using the expression obtained by symbolic regression to approximate the oviposition rate.

To validate the reasonableness of the functional form obtained through symbolic regression, we integrated the oviposition rate function (5) into model (1) to establish and solve a traditional dynamics model. The comparison of the simulation results obtained by approximating oviposition rates using the neural network and function (5) respectively is shown in Fig 10. Although the two approaches use different underlying principles, their simulation results are mostly consistent. This demonstrates that function (5) closely aligns with the neural network’s performance. Moreover, utilizing function (5) to construct a mechanistic model effectively captures the changes in mosquito populations. These findings validate the suitability and efficacy of the obtained functional form for oviposition rate.

### Comparison and analysis of oviposition rate functions

In this section, we compared and discussed the function we obtained with various forms used in earlier research. The forms of oviposition rates in previous studies can be categorized into four main types: constant, temperature-dependent, humidity-dependent, and dependent on both temperature and precipitation, as shown in Table 4.

**Table 4:** Comparison of oviposition rate functions.

Variable	Function	Source
Constant	—	Erickson [18], Tran [25]
Temperature	$\begin{array}{l} B(T)=b_{0}+b_{1}\times T+b_{2}\times T^{2}+b_{3}\times T^{3}+b_{4}\times T^{4}\\ B(T)=b_{0}+b_{1}\times T+b_{2}\times T^{2}+b_{3}\times T^{3} \end{array}$	Yang [28], Jia [9], Fukui [17]
Temperature	$B(T)=ae^{-b{\left(\frac{T-T_{b}}{v_{b}}\right)}^{2}{\left(T_{d}-T\right)}^{1.5}}$	Metelmann [51], Wang [64]
Humidity	$B(W)=\frac{E_{\max}}{1+e^{-\frac{W-E_{mean}}{E_{\mathrm{var}}}}}+b_{0}$	Gong [21]
Temperature, precipitation	$\begin{array}{c} B(T,R)=\alpha u(T)v(T)\\ u(T)=e^{-a{\left(T-T_{b}\right)}^{2}},v(R)=\frac{(1+s)e^{-r{\left(R-R_{b}\right)}^{2}}}{e^{-r{\left(R-R_{b}\right)}^{2}}+s} \end{array}$	Abdelrazec [65], Liu [66]

Temperature-dependent functions primarily originate from the works of Yang et al. and Metelmann et al. [28,51], who fitted data from controlled temperature experiments in laboratories using forms such as 4th-degree polynomials and skewed Gaussian probability density functions. Experimental data indicate a quasi-linear increase in oviposition rates with rising temperature. The oviposition rates we obtained also demonstrate a similar trend when fixing precipitation. However, the experiments observed a decrease in oviposition rate at extreme temperatures, which was not captured by our model. Several factors may account for this difference. Laboratory conditions are tightly controlled, whereas natural environmental factors such as temperature and precipitation exhibit coordinated variability, highlighting significant differences between these environments. Therefore, in the actual ecological setting of Guangdong Province, this decline may not necessarily manifest. Furthermore, due to the limited sample size of extreme weather conditions in Guangdong, further research is needed to determine whether extreme temperatures lead to decreased oviposition rates under real ecological conditions.

Humidity-dependent function was proposed by Gong [21]. Although his research focused on the Culex mosquito, the oviposition rate was proposed based on the broader behavioral patterns of mosquito populations. Based on the effect of rainfall on mosquito dynamics, Gong hypothesized that there is a positive correlation between oviposition rate and humidity index and formulated it through a sigmoid function. The oviposition rate computed by this function increases exponentially with rising humidity, gradually reaches saturation, and then remains stable. However, this function’s independent variable is humidity, whereas our function’s independent variables are temperature and precipitation. The difference in the independent variables leads to the incomparability of the two functions. However, a simple analysis of oviposition rate trends can be conducted. Gong’s findings indicate that rainfall positively impacts mosquito oviposition. But some scholars [12] argue that excessive precipitation may negatively affect oviposition, disrupting activities such as blood feeding, host seeking, and the search for oviposition sites. Our results show a decrease in oviposition rates with excessive precipitation, supporting this viewpoint. Additionally, if we regard the humidity index as a function of precipitation and temperature, then our function closely resembles Gong’s function in form.

In addition, a limited number of scholars have indeed considered both temperature and precipitation in the oviposition rate function. For instance, Abdelrazec et al. [65] separately modeled the effects of temperature and precipitation and then multiplied these functions to represent their combined impact. After selecting appropriate parameters, this method shows a nearly linear increase in oviposition rate with rising temperatures. At a constant temperature, oviposition rates exhibit an initial increase followed by a decrease with increasing precipitation, which is broadly consistent with our results. However, this approach has limitations because it assumes temperature and precipitation act independently in the oviposition process. Under this formulation, the optimal precipitation level remains constant across different temperatures, and the optimal temperature remains constant across varying precipitation conditions, which contradicts biological knowledge. In contrast, our model incorporates interaction terms between temperature and precipitation, demonstrating that their effects on oviposition rates are interdependent, which aligns more closely with biological principles.

In summary, the trends of oviposition rate with changes in temperature and precipitation in our study closely align with previous research findings. Our function integrates the combined effects of temperature and precipitation on oviposition rate, revealing their complex interactions that better reflect biological behaviors. Our research can serve as a reference for future studies and offer new perspectives on the application of neural networks in biological dynamics.

## Discussion and conclusion

Integrating ideas derived from machine learning with traditional engineering-related methods facilitates the incorporation of domain knowledge and known physical information into the learning process [67]. Recent studies have shown the feasibility of merging differential equations with neural networks [34]. For example, Dandekar et al. developed an SIR model for disease transmission and integrated neural networks to quantify the impact of government control measures [38]. However, this approach has not received significant attention. In fact, coupling neural networks with mechanistic models and using neural networks to explore unknown mechanisms is highly valuable. In this paper, we apply this concept to population dynamics, focusing on using a coupled model of mechanistic model and neural network to simulate changes in mosquito population abundance and explore the relationships between oviposition behavior, temperature, and precipitation. This approach allows for more accurate simulations of mosquito dynamics while also enhancing the biological interpretability of our model.

In this work, we first constructed a two-stage mosquito model based on the life cycle, including immatures and adults. This approach is consistent with traditional dynamics models, with differential equations describing intrinsic mechanisms such as growth and reproduction. Secondly, recognizing that the oviposition process is influenced by both temperature and precipitation, yet existing studies rarely offer a specific function to describe this relationship, we employed a neural network to approximate the oviposition function and embedded it into the dynamic equations. This approach resulted in a coupled model that combines the strengths of both neural networks and traditional mechanistic models. Subsequently, data from nine cities in Guangdong Province were used for model training, and data from three cities were used as testing sets for model verification, which allowed us to obtain a set of biological and network parameters applicable to all cities, ensuring the model’s generalizability.

The simulated results of our model closely aligned with the observed data. Compared with previous studies [9,19,31,51], our model simultaneously fitted data from nine cities, with correlation coefficients above 0.7 for all cities (shown in Figs 5 and 6). Particularly in Heyuan, Shenzhen, and Guangzhou, the coefficients exceed 0.85, indicating highly satisfactory simulation performance. Moreover, evaluations on the testing set reveal correlation coefficients exceeding 0.8 for Zhuhai and Huizhou, further confirming the robustness and effectiveness of the model. However, deviations between simulated and actual values are noticeable in some localized positions. In fact, from a modeling perspective, while neural networks can enhance predictive capabilities, attempting to fit data from all cities with the same set of parameters inevitably requires sacrificing some local fitting accuracy to achieve better overall fitting performance. Additionally, several factors contribute to deviations in simulation results. Since the initial number of immature mosquitoes cannot be precisely determined, we followed Tran’s initial value settings [25] and advanced the simulation start time to 2015. Nevertheless, this adjustment may still impact simulation results in 2016. Furthermore, inherent uncertainties and stochastic fluctuations in observational data also affect the model’s performance. Variations in the standardization and accuracy of data monitoring procedures among different regions contribute to heightened data uncertainty. Moreover, differences in the timing and intensity of mosquito control measures implemented by various cities in response to population increases could affect mosquito data, thereby influencing the effectiveness of our model.

The inputs and outputs of the neural network revealed the relationship between oviposition rate, temperature, and precipitation (shown in Figs 7 and 8). Under varying levels of precipitation, the trend of oviposition rate with temperature changes closely resembled an S-shaped curve, indicating that optimal temperatures promote mosquito egg-laying, while excessively high temperatures may have adverse effects. The impact of precipitation on the oviposition rate is also nonlinear, with the oviposition rate exhibiting a parabolic trend in response to precipitation changes. Optimal precipitation levels provide more breeding sites, stimulating mosquito egg-laying. However, excessive precipitation may negatively impact oviposition. Both low and high levels of precipitation can alter the threshold temperature required for a significant increase in mosquito oviposition rates. Additionally, different temperatures may also influence the precipitation levels needed for the oviposition rate to reach its peak and then decline from its maximum.

In addition, based on the results of the neural network, we employed symbolic regression to search for the optimal expression of the oviposition rate that depends on temperature and precipitation. The expression we obtained closely corresponded to the neural network trained previously (shown in Fig 9). The simulation results of the mechanistic model established using this oviposition rate function were highly consistent with our prior simulations and aligned well with the trends of the MOI, validating the effectiveness of the symbolic regression results. Furthermore, we compared and discussed the obtained expression with those used in previous studies. In contrast, our function integrates both temperature and precipitation factors affecting oviposition, emphasizing their coordinated influence and thereby providing a more accurate reflection of biological behaviors in practice.

Recently, deep neural networks have shown remarkable effectiveness in addressing traditionally challenging problems such as image recognition and natural language processing. In studies on population dynamics of mosquitoes, some scholars have achieved promising results using machine learning methods. However, these studies often directly employ factors such as temperature, precipitation, and sunlight as inputs, with mosquito abundance as the output, heavily relying on data for model training. The complexity and multidimensionality of these algorithms cause models to function as black-boxes, which makes it challenging to interpret the various physiological mechanisms involved in the simulation process. Traditional population dynamics models describe a range of biological mechanisms, but the functional forms involved such as oviposition rate, development rate, and mortality rates must be predetermined, which significantly limits the performance of dynamics models. The highlight of our model lies in the coupling of the mechanistic model and the neural network, which allows it to adhere to biological mechanisms while mining trends in data, thus effectively overcoming these limitations. This method also allows us to capture information about reproduction, development, and other processes directly from data. Consequently, for less understood mechanisms, we can approximate them using neural networks, without the need for predefined functions. This coupling approach enhances the biological interpretability of the model and provides new insights into the dynamics of complex systems.

In summary, we developed a modeling method for population dynamics that integrates mechanistic models with machine learning techniques, and accurately modeled the trends of Aedes mosquitoes. Our study also explored how the oviposition rate is influenced by the combined effects of temperature and precipitation and provided a precise mathematical expression. However, our model also has some limitations. Firstly, the Aedes mosquito data we have collected actually includes information on both Aedes albopictus and Aedes aegypti, and we are unable to distinguish data for each species individually. Since these two species have different thermal responses, exploring the specific effects of temperature and rainfall on each mosquito species still requires further research. Secondly, in natural environments, excessive rainfall can cause a decrease in temperature due to the interconnection between the two factors. As a result, scenarios with both high rainfall and relatively high temperatures are less common, so the network’s learning outcomes should be treated as a reference. Additionally, our set of diapause is relatively simple, and we did not consider the effects of human interventions, such as government mosquito control measures. We intend to address these aspects in future work, but we believe that this method, combining dynamics models with deep learning, holds potential for broader applications.
